# Inhibition of Heat Shock Protein 90 by 17-AAG Reduces Inflammation via P2X7 Receptor/NLRP3 Inflammasome Pathway and Increases Neurogenesis After Subarachnoid Hemorrhage in Mice

**DOI:** 10.3389/fnmol.2018.00401

**Published:** 2018-11-06

**Authors:** Yuchun Zuo, Jikai Wang, Fan Liao, Xiaoxin Yan, Jianming Li, Lei Huang, Fei Liu

**Affiliations:** ^1^Department of Neurosurgery, Third XiangYa Hospital, Central South University, Changsha, China; ^2^Department of Anatomy, XiangYa Medical School, Central South University, Changsha, China; ^3^Neuroscience Research Center, Changsha Medical University, Changsha, China; ^4^Department of Physiology and Pharmacology, School of Medicine, Loma Linda University, Loma Linda, CA, United States; ^5^Department of Neurosurgery, School of Medicine, Loma Linda University, Loma Linda, CA, United States

**Keywords:** subarachnoid hemorrhage, 17-AAG, heat shock protein 90, purinoceptor, NLRP3 inflammasome, neurogenesis, P2X7 receptor

## Abstract

Subarachnoid hemorrhage (SAH) is a life-threatening cerebrovascular disease that usually has a poor prognosis. Heat shock proteins (HSPs) have been implicated in the mechanisms of SAH-associated damage, including increased inflammation and reduced neurogenesis. The aim of this study was to investigate the effects of HSP90 inhibition on inflammation and neurogenesis in a mouse model of experimental SAH induced by endovascular surgery. Western blotting showed HSP90 levels to be decreased, while neurogenesis, evaluated by 5-bromo-2’-deoxyuridine (BrdU) immunohistochemistry, was decreased in the hippocampuses of SAH mice. SAH also induced pro-inflammatory factors such as interleukin-1β (IL-1β), capase-1 and the NLRP3 inflammasome. However, intraperitoneal administration of the specific HSP90 inhibitor 17-allylamino-17-demethoxygeldanamycin (17-AAG) reduced the levels of HSP90, NLRP3, ASC, caspase-1 and IL-1β, while increasing the levels of brain-derived neurotrophic factor and doublecortin (DCX), as well as the number of BrdU-positive cells in SAH mice. In addition, 17-AGG improved short- and long-term neurobehavioral outcomes. The neuroprotective and anti-inflammatory effects of 17-AGG were reversed by recombinant HSP90 (rHSP90); this detrimental effect of HSP90 was inhibited by the specific P2X7 receptor (P2X7R) inhibitor A438079, indicating that SAH-induced inflammation and inhibition of neurogenesis were likely mediated by HSP90 and the P2X7R/NLRP3 inflammasome pathway. HSP90 inhibition by 17-AAG may be a promising therapeutic strategy for the treatment of SAH.

## Introduction

Subarachnoid hemorrhage (SAH) is a devastating and life-threatening cerebrovascular disease with high morbidity and mortality and remains a serious problem worldwide. This stroke subtype is up to 10%–15% fatality in patients before they reach to hospital (Nichols et al., 2018). Although increasingly early diagnosis with brain imaging and neurosurgical interventions are available, 8%–20% of the victims were permanently disabled (van Gijn et al., 2007; Nieuwkamp et al., 2009). SAH mainly affects middle-aged patients and leads to disability, resulting in a high socioeconomic burden (Lapchak and Zhang, 2017).

Neuronal death is the principal consequence of SAH-associated pathology. Although neurogenesis was once thought to be absent in the adult brain, it has recently been shown to occur continuously in the subgranular (SGZ) and subventricular (SVZ) zones of the hippocampal dentate gyrus (DG) and the walls of the lateral ventricles, respectively (Arai et al., 2011). As a part of neurovascular remodeling, neurogenesis mainly occurs days to weeks after stroke, depending on the crosstalk between neurons, astrocytes, endothelial cells and glia (Xing et al., 2012). Brain-derived neurotropic factor (BDNF) is one of the most important trophic factors promoting neurogenesis when present in the microenvironment. BDNF has been shown to play essential roles in inducing neuronal progenitor cell proliferation and angiogenesis, promoting blood-brain barrier (BBB) reconstruction and tissue remodeling, and improving neurological function after brain injury (Kromer, 1987; Nagahara and Tuszynski, 2011; Xing et al., 2012; Grande et al., 2013).

Inflammation, one of the main causes of early brain injury (EBI), is considered a key modulator of neurogenesis (Borsini et al., 2015). In addition, inflammation is considered a major contributor to multiple neurological disorders that are mainly associated with hippocampus dysfunction. Interleukin-1β (IL-1β) is the predominant pro-inflammatory cytokine induced by brain injury and the pro-inflammatory microenvironment changes are known to inhibit neural precursor cell (NPC) proliferation in both embryonic and adult hippocampuses (Ryan et al., 2013). The NLRP3 inflammasome is the main inducer of IL-1β production and, along with other pro-inflammatory factors, has been established to contribute to the pathophysiology of EBI (Chen et al., 2013; Khalafalla et al., 2017; Zhou et al., 2017). The P2X7 receptor (P2X7R), which is abundant in the central nervous system and widely expressed in neurons and glial cells, is a ligand-gated non-selective cation channel in which activation is associated with inflammation and oxidative stress (Monif et al., 2009; Di Virgilio et al., 2017). The P2X7R, when stimulated by ATP, causes the flux of Na^+^, Ca2^+^ and K^+^; therefore, membrane depolarization occurs due to these ionic flux induced electrochemical gradients (North, 2002). The activation of P2X7R has been shown to induce several key inflammatory molecules, including IL-1β production; this P2X7R-dependent cytokine production is driven by activating NLRP3 inflammasome (Deplano et al., 2013; Khalafalla et al., 2017). Moreover, a previous study showed that the P2X7R/inflammasome axis is involved in neuroinflammation after SAH (Chen et al., 2013).

Heat shock protein 90 (HSP90) mediates protein folding and maturation and is thus considered to maintain cellular protein homeostasis (Wang et al., 2018). Numerous studies have shown that HSP90 inhibition exerts neuroprotective effects by attenuating necroptosis and inflammation and by preserving the BBB after ischemic stroke (Qi et al., 2014, 2015; Wang et al., 2018). Interestingly, a recent study showed HSP90 to be essential for P2X7R stabilization and function (Migita et al., 2016). Tanespimycin 17-allylamino-17-demethoxygeldanamycin (17-AAG) is a potent specific chemical inhibitor of HSP90. 17-AAG has been widely used to study the function of HSP90, and is currently being assessed in a series of clinical trials, most of which are focusing on its antitumor effects (Kamal et al., 2003; Lv et al., 2018; Ma et al., 2018). In contrast, the neuroprotective effect of 17-AAG has been shown in ischemic stroke animal models to have anti-inflammation effects and protect neural progenitor cells from death (Bradley et al., 2014; Li et al., 2015). Therefore, it is rational that HSP90, as an essential factor of P2X7R activation, may be involved in the pathology of hemorrhagic stroke via SAH, and that 17-AAG may exert its neuroprotective potential by targeting HSP90, thereby inhibiting the P2X7R/NLRP3 inflammasome axis and promoting neurogenesis.

The goal of the present study was to determine if administration of 17-AAG could exert neuroprotective effects in a mouse model of experimental SAH. Specifically, we hypothesized that inhibition of HSP90 by 17-AAG could reduce inflammation through the P2X7R/NLRP3 inflammasome pathway and increase neurogenesis following SAH-inducing surgery. In the present study, we explored the possible neuroprotective effects of 17-AAG in a SAH mouse model. Our results demonstrated that 17-AAG causes anti-inflammation and pro-neurogenesis effects, thereby improving neurobehavioral function.

## Materials and Methods

### Animals and the Experimental SAH Procedure

Adult male KM mice (8 weeks old) were purchased from Slac Corporation (Changsha, China). Mice were housed at a constant temperature (25°C) in a humidity-controlled room under a 12-h light/dark cycle and had access to food and water *ad libitum*. All experimental procedures and protocols were approved by the Ethics Committee of Central South University and performed according to the Guide for the Care and Use of Laboratory Animals of the National Institutes of Health (eighth edition) and reported according to the ARRIVE guidelines.

Experimental SAH was induced in mice using an endovascular perforation technique described previously (Muroi et al., 2014; Siler et al., 2014). Briefly, after carefully exposing the carotid artery, a nylon suture (5–0) was inserted into the external carotid artery, advanced via the internal carotid to the bifurcation of the anterior and middle cerebral arteries, and then forced slightly further to induce a hemorrhage. Afterward, the nylon suture was removed. In sham-operated animals the suture was inserted and advanced without arterial perforation.

### Experimental Design

The experimental designs are schematically shown in Supplementary Figure S1.

#### Experiment 1: Characterization of the Temporal Pattern of HSP90 Expression

Thirty mice were divided randomly into five groups (*n* = 6 per group): sham and SAH 12, 24, 48 and 72 h. Neurological function was measured by the modified Garcia score and beam balance test. Mice were euthanized at different time points after SAH. The SAH grade was measured during sample collection. Another eight mice were injected intraperitoneally (i.p.) with 5-bromo-2’-deoxyuridine (BrdU; 50 mg/kg in saline, Sigma Aldrich, St. Louis, MO, USA) were used for immunohistochemistry staining in the sham and SAH groups (*n* = 4 per group).

#### Experiment 2: Evaluation of the Neuroprotective Effects of 17-AAG

The HSP90 inhibitor 17-AAG (MedChemExpress, Princeton, NJ, USA) was i.p. administered 1 h after the SAH procedure. Twenty-four mice were divided randomly into four groups (*n* = 6 per group): sham, SAH + vehicle, SAH + 17-AAG (27 mg/kg) and SAH + 17-AAG (80 mg/kg). The modified Garcia score, beam balance test, and brain water content were used to evaluate the effects of 17-AAG administration.

#### Experiment 3: Identification of Potential Mechanisms of 17-AAG-Mediated Anti-inflammatory and Pro-neurogenesis Effects

Mice were randomly divided into five groups (*n* = 10 per group): sham, SAH + vehicle, SAH + 17-AAG, SAH + 17-AAG + vehicle, SAH + 17-AAG + recombinant HSP90 (rHSP90). Treatment with vehicle/17-AAG (80 mg/kg, i.p.) was performed 1 h after SAH, whereas rHSP90 (150 μg/kg; Cusabio, China) was administered by intra-cerebroventricular (i.c.v.) injection 1 h before SAH induction. Western blotting was performed to detect protein levels. BrdU labeling of nuclei was used to evaluate neurogenesis. BrdU was i.p. injected 0.5 h after SAH. BrdU and doublecortin (DCX) immunohistochemical staining and quantitative analyses were performed 48 h after SAH induction. Besides, another 12 mice underwent sham operation were divided into two groups (*n* = 6 per group): sham + vehicle, sham + 17-AAG. Mice underwent sham operation administered vehicle or 17-AAG were employed to test if the 17-AAG could affect the proteins level related to the pathway under sham condition.

#### Experiment 4: Confirmation of the 17-AAG Mechanism of Action

To confirm the HSP90-mediated mechanism of 17-AAG-induced effects, A438079 (15 mg/kg; MedChemExpress, Princeton, NJ, USA), an inhibitor of P2X7R, and rHSP90 were administered 1 h before (i.p.) and 1 h after (i.c.v.) SAH, respectively. Mice were randomly divided into five groups (*n* = 10 per group): sham, SAH + vehicle, SAH + rHSP90, SAH + rHSP90 + vehicle and SAH + rHSP90 + A438079. Immunoblotting was performed to detect proteins levels. BrdU was i.p. injected to label the nuclei of proliferating cells. BrdU and DCX immunohistochemical staining and quantitative analyses were performed 48 h after SAH.

#### Experiment 5: Evaluation of Long-Term Behavioral Effects of 17-AAG

To assess the long-term effects of 17-AAG on neurobehavioral functions after SAH, 18 mice were randomly divided into three groups (*n* = 6 per group): sham, SAH + vehicle and SAH + 17-AAG (80 mg/kg). The Rotarod and Morris water maze tests were performed on days 7, 14 and 21 and days 21–25 after SAH, respectively.

#### Experiment 6: Demonstration of P2X7R Involvement

To further confirm that P2X7R is involved in the SAH-induced signaling pathway, A438079 was i.p. administered 1 h after SAH. Thirty mice were randomly divided into three groups (*n* = 10 per group): sham, SAH + vehicle and SAH + A438079. Western blotting was performed to detect the protein levels of the signaling pathway components. Immunohistochemistry for BrdU and DCX was performed, and BrdU- and DCX-positive cells were quantified 48 h after SAH.

### I.c.v. Injection

I.c.v. injection was performed as described previously (Iniaghe et al., 2015; Chen et al., 2018). Briefly, a cranial burr hole was drilled 0.3 mm posterior and 1.0 mm lateral to Bregma. A 10-μL Hamilton syringe with a 26-gauge needle was used for drug administration. The needle was inserted at a depth of 2.3 mm into the right lateral ventricle through the burr hole. The rate of i.c.v. injection was 0.5 μL/min. The needle was left in place for an additional 8 min after the end of infusion and then slowly removed over a period of 3 min. The burr hole was sealed with bone wax.

### SAH Grade Measurement

The severity of SAH was evaluated according to a previously described grading system (Altay et al., 2014). Briefly, the system is based on the amount of subarachnoid blood clots distributed in the six segments of the basal cistern: 0, no subarachnoid blood; 1, minimal subarachnoid clots; 2, moderate subarachnoid clots with recognizable arteries; and 3, blood clots covering all arteries. A total score, ranging from 0 to 18, was obtained by adding the scores of all six segments. The grading was performed by a researcher blinded to the experiment.

### Assessment of Neurological Function

Short-term neurological function was assessed using the modified Garcia scoring system and the beam balance test 24 and 48 h after SAH induction, as previously described (Sugawara et al., 2008). The Garcia score was composed of six test subscores, evaluating deficits in spontaneous activity, spontaneous movement of the four limbs, forepaw outstretching, climbing, body proprioception, and whisker proprioception. A total score, ranging from 3 to 18, was obtained by adding the six test subscores, with higher values indicating better function (Liu et al., 2018). Beam balance tests evaluated the ability of animals to walk on a narrow cylindrical wooden beam. The animals were placed on the beam and allowed to walk freely for 60 s. The score was evaluated as follows: 0, no walking and falling; 1, no walking, but remains on the beam; 2, walking but falling; 3, walking less than 20 cm; 4, walking beyond 20 cm. The assessment was performed by a researcher who was blinded to the experiment. Long-term neurobehavioral effects were assessed using the Rotarod test, which examines the abilities of balance and sensorimotor coordination, 1, 2 and 3 weeks following SAH. Performance in the Morris water maze was used to evaluate the abilities of spatial learning and memory on days 21–25 after SAH, as previously reported (Xie et al., 2018).

### Brain Water Content

Brain water content was evaluated by a wet/dry method, as described previously (Liu et al., 2015). The brains were divided into four parts (left hemisphere, right hemisphere, cerebellum, and brain stem) and weighed immediately (wet weight), then weighed again (dry weight) after incubation at 100°C for 72 h. The formula for brain water content calculation was [(wet weight − dry weight)/wet weight] × 100%.

### Western Blot Analysis

Western blotting was performed as described previously (Xiao et al., 2018). Briefly, brain tissues were extracted in RIPA buffer (Beyotime, Shanghai, China), and the protein concentrations were measured using a bicinchoninic acid kit (Beyotime, Shanghai, China). Protein samples were separated by 8%–12% SDS-PAGE and transferred onto polyvinylidene fluoride membranes (Millipore, Burlington, MA, USA). After being blocked with 5% nonfat milk for 1 h, the membranes were incubated overnight at 4°C with the following primary antibodies: anti-HSP90 (1:1,000; Proteintech, China) anti-P2X7R (1:1,000; Santa Cruz Biotechnology, Santa Cruz, CA, USA), anti-NLRP3 (1:500; Abcam, Cambridge, MA, USA), anti-ASC (1:500; Santa Cruz Biotechnology, Santa Cruz, CA, USA), anti-caspase-1 (p20; 1:1,000; Abcam, Cambridge, MA, USA), anti-IL-1β (1:1,000; Proteintech, China) anti-BDNF (1:1,000; Abcam, Cambridge, MA, USA), anti-DCX (1:1,000; Abcam, Cambridge, MA, USA), anti-caspase-3 (1:1,000; Cell Signaling Technology, Danvers, MA, USA), anti-HSP70 (1:1,000; Proteintech, China), anti-HSP40 (1:1,000; Proteintech, China) and anti-GAPDH (1:2,000; Proteintech, China). After incubation with the appropriate secondary antibody, the specific bands were visualized by enhanced chemiluminescence (Super Signal Pierce Biotechnology). The relative densities of the bands were analyzed using ImageJ software.

### Immunohistochemistry

Immunohistochemistry was performed as previously described (Muroi et al., 2014). Briefly, 20-μm coronal brain sections were cut using a cryostat (Leica CM3050S, Buffalo Grove, IL, USA). For immunohistochemical detection of BrdU-labeled nuclei, sections were pre-treated with 50% formamide/2× SSC at 65°C for 1 h, and the DNA was denatured in 2 N HCl for 30 min. The sections were incubated with the following primary antibodies overnight at 4°C: anti-HSP90 (1:1,000; Proteintech, China), anti-BrdU (1:1,000; Abcam, Cambridge, MA, USA), or anti-DCX (1:1,000; Abcam, Cambridge, MA, USA). The sections were then incubated with a biotinylated secondary antibody (1:400; Abcam, Cambridge, MA, USA) for 1 h and with ABC reagents (1:400; Vector Laboratories Inc., Burlingame, CA, USA) for an additional hour, following which the immunoreactivity was visualized using 0.003% H_2_O_2_ and 0.05% 3, 3′-diaminobenzidine.

### Immunofluorescence Detection

Double immunofluorescence staining was performed as described previously (Xiao et al., 2018). Briefly, after blocking with 5% donkey serum for 1 h, tissue slices were incubated overnight at 4°C with the following primary antibodies: rabbit anti-HSP90 (1:100; Proteintech, China), mouse anti-HSP90 (1:100; Proteintech, China), rabbit anti-Iba-1 (1:100; Proteintech, China), mouse anti-NeuN (1:200; Abcam, Cambridge, MA, USA), goat anti-DCX (1:200; Abcam, Cambridge, MA, USA), and rat anti-BrdU (1:200; Abcam, Cambridge, MA, USA), followed by incubation with the appropriate fluorophore-conjugated secondary antibody. The sections were examined under an Olympus BX51 fluorescence microscope.

### Immunoprecipitation (IP) Detection

The left hemisphere was harvested and lysed in cold IP extraction buffer, following centrifuged at 12,000 rpm at 4°C for 20 min. Five microgram primary antibodies were pre-incubated with 30 μl agarose-G for 5 h at room temperature, then rinsed with GLB+ buffer for five times. Thereafter, brain lysate was incubated with agarose-G combined with primary antibody at 4°C for 24 h. Then, the proteins washed with cold GLB + buffer three times, followed by eluted with prepared 1× loading buffer in boiling water for 8 min and centrifuged at 12,000 rpm for 2 min. The supernatant was collected and loaded to SDS–PAGE.

### Quantification and Statistical Analysis

The number of BrdU-labeled cells in the DG of hippocampal sections was counted in each animal in each of three coronal sections (20-μm apart) in a high-power field (400× magnification) using an Olympus BX51 microscope with digital camera. The hippocampal sections were employed to do the quantification analyses, the number of BrdU immunolabeling nuclei were counted in the area of DG, which including the hilus, SGZ, and granule cell layer (GCL) as previously described (Mino et al., 2003). Results were recorded as the average number of BrdU-positive cells per section and reported as the mean ± SD. The densities of the regions of interest were measured using ImageJ software after a background subtraction. The data were expressed as means ± SD. All data were plotted using GraphPad Prism 7 (Graph Pad Software, San Diego, CA, USA). SPSS 16.0 software (SPSS Inc., Chicago, IL, USA) was used for statistical analysis. One-way analysis of variance (ANOVA) followed by Tukey’s *post hoc* test was used for multiple comparisons among groups. Two-way ANOVA was applied to analyze long-term neurobehavioral results. Statistical significance was defined as *P* < 0.05.

## Results

### General Observations and SAH Severity

A total of 226 mice were used in this study (Supplementary Figure S1). Thirty-four mice underwent sham surgery and 192 mice underwent SAH surgery. The average SAH grades among the SAH groups showed no statistical differences. The mortality rate of SAH mice was 21.3% (40 of 188; four mice were excluded from the study because of mild SAH (SAH grade <7). The mortality rate showed no statistically significant differences among the SAH groups. No mice in the sham group died.

### Temporal Pattern of HSP90 Expression in the Left Hemisphere After SAH

**Figure 1:**
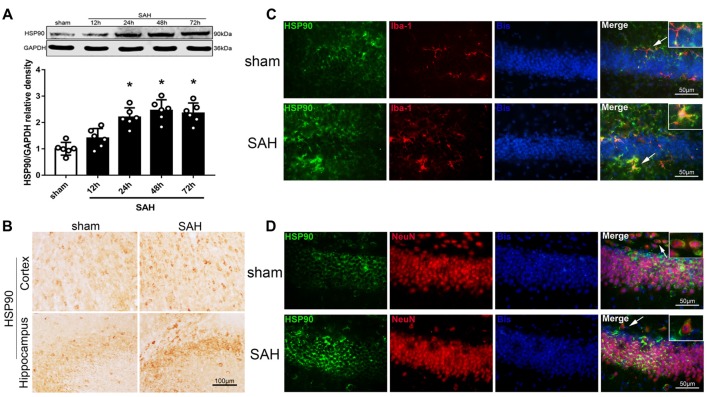
Expression of heat shock protein 90 (HSP90) after subarachnoid hemorrhage (SAH). **(A)** Representative western blot images and quantitative analyses of the HSP90 time course in the left hemisphere after SAH; *n* = 6 per group. **(B)** Immunohistochemistry staining for HSP90 in the left cortex and hippocampus. **(C)** Double immunofluorescence staining for HSP90 (green) in microglia (Iba-1, red) in the left hippocampus 48 h after surgery. **(D)** Double immunofluorescence staining for HSP90 (green) in neurons (NeuN, red) in the left hippocampus 48 h after surgery; *n* = 4 per group. *P < 0.05 vs. sham group. The error bars represent SD. Bis, bisbenzimide; Iba-1, ionized-calcium-binding adaptor molecule-1; NeuN, neuronal nuclear.

We used western blotting to detect the expression of HSP90 in the left hemisphere 12, 24, 48 and 72 h after SAH (Experiment 1). The expression of HSP90 significantly increased starting 24 h after SAH and peaked at 48 h (Figure 1A). The HSP90 protein levels 48 h post-SAH were nearly 2.5 times higher than those in the sham group (Figure 1A). Immunohistochemistry was performed to detect HSP90 in the left cortices and hippocampuses of SAH mice (Figure 1B). Double immunofluorescence staining showed that HSP90 was localized in both the microglia (Iba-1-positive cells) and neurons (NeuN-positive cells) of the hippocampus 48 h after SAH (Figures 1C,D). The time course of HSP70 and HSP40 were also detected. The results showed the expression of HSP70 was significantly increased over 24–72 h after SAH, while the HSP40 protein level were not changed (Supplementary Figure S2).

### BrdU-Positive Progenitor Cells and DCX Expression in the Left Hippocampus After SAH

**Figure 2:**
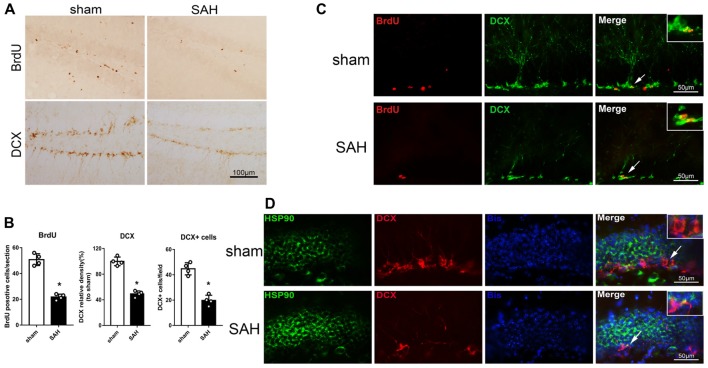
Expression of doublecortin (DCX) and 5-bromo-2’-deoxyuridine (BrdU) incorporation after SAH. **(A)** Immunohistochemistry staining and **(B)** quantitative analyses of BrdU and DCX in the left hippocampus in sham and SAH groups at 48 h. **(C)** Double immunofluorescence staining for BrdU (red) and DCX (green) in the left hippocampus 48 h after surgery. **(D)** Double immunofluorescence staining for HSP90 (green) and DCX (red) in the left hippocampus at 48 h after surgery; *n* = 4 per group. *P < 0.05 vs. sham group. The error bars represent SD.

Immunohistochemistry was performed to detect BrdU and DCX in the left hippocampus after SAH (Figure 2A). The BrdU and DCX levels 48 h after SAH were lower than in the sham group (Figure 2B). Immunofluorescence staining showed DCX expression in immature neurons/NPCs (BrdU-positive cells) in the hippocampus (Figure 2C). Additionally, HSP90 co-localized with DCX in the hippocampus 48 h after SAH (Figure 2D). The results suggested the hippocampal neurogenesis was impaired at 48 h after SAH, which may involve HSP90 signaling.

### HSP90 Inhibitor 17-AAG Improves Short- and Long-Term Neurobehavioral Performance and Reduces Brain Edema After SAH

**Figure 3:**
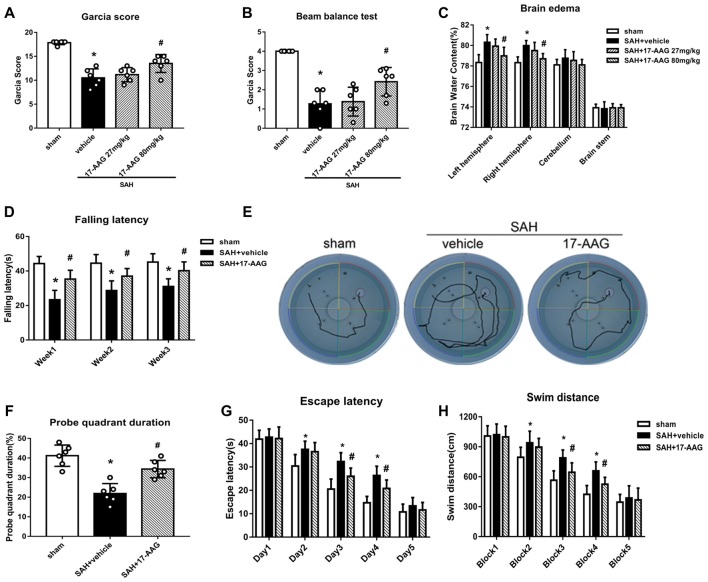
HSP90 inhibitor 17-allylamino-17-demethoxygeldanamycin (17-AAG) improved short- and long-term neurobehavioral outcomes of SAH. **(A)** Modified Garcia scores and **(B)** beam balance test scores 48 h after SAH. **(C)** Brain water content; *n* = 6 per group. **(D)** Rotarod test results. **(E)** Trace map of the Morris water maze test. **(F)** Time spent in the probe quadrant, **(G)** escape latency and **(H)** swimming distance in the water maze test; *n* = 6 per group. *P < 0.05 vs. sham group; ^#^*P* < 0.05 vs. SAH + vehicle group. The error bars represent SD.

The modified Garcia and beam balance tests produced significantly lower scores in the SAH + vehicle group than in the sham group (Experiment 2). Administration of a high dose of 17-AAG (80 mg/kg) significantly improved the neurological scores compared with vehicle treatment (*P* < 0.05; Figures 3A,B). The brain edema is the one of main pathophysiology after SAH, which related to mortality and poor outcome. A dry/wet weigh method was applied to detect the brain edema by 17-AAG treatment after SAH. The brain water content was significantly increased in both hemispheres in the SAH + vehicle group compared with that in the sham group (*P* < 0.05, Figure 3C). However, the brain edema was significantly attenuated by a high dosage of 17-AAG (80 mg/kg; *P* < 0.05, Figure 3C). The brain water contents in the cerebellum and brain stem showed no significant differences between the sham and SAH groups. Based on the short-term neurological outcomes and brain water content results, we chose the high dose of 17-AAG for the long-term and mechanistic studies.

Balance and sensorimotor coordination functions were measured by the Rotarod test (Experiment 5). The SAH + vehicle group had a significantly shorter falling latency compared with that of the sham group. However, 17-AAG treatment improved the performance significantly 1 to 3 weeks post-SAH (*P* < 0.05; Figure 3D).

Spatial learning and memory functions were measured by the Morris water maze test (Experiment 5). The rats with spatial learning and memory deficits usually swim more and take longer time to find the platform submerged in the water. The SAH + vehicle group showed a longer swimming distance to find the platform (Figures 3E,H), as well as a longer escape latency, than the sham group. However, both parameters were significantly improved by 17-AAG treatment on the performance on days 3 and 4 (*P* < 0.05; Figure 3G) and blocks 3 and 4 in the SAH + 17-AAG group (*P* < 0.05; Figure 3H). Probe trials were conducted with the platform removed, and the percentage of time spent in the target quadrant was notably decreased in SAH + vehicle group compared with that in the sham group. However, 17-AAG treatment significantly increased the time SAH mice spent in the probe quadrant (*P* < 0.05; Figure 3F). These findings suggested the 17-AAG improved SAH induced spatial learning and memory deficits at 4 weeks after SAH.

Taken together, the results of this section showed that 17-AAG (80 mg/kg) improved both short- and long-term neurological deficit, and reduces brain edema after SAH.

### Effect of 17-AAG on Neurogenesis 4 Weeks After SAH

**Figure 4:**
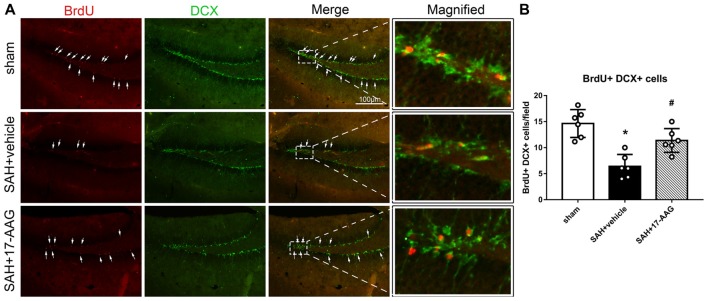
Effects of 17-AAG on neurogenesis 4 weeks after SAH. **(A)** Double immunofluorescence staining and **(B)** Quantitative analyses of BrdU^+^ DCX^+^ cells in the left hippocampus 4 weeks after SAH. *n* = 6 per group *P < 0.05 vs. sham group; ^#^*P* < 0.05 vs. SAH + vehicle group. The error bars represent SD.

Double immunofluorescence staining of BrdU and DCX, widely used to label new borne neuronal progenitors, was performed to detected the neurogenesis 4 weeks after SAH (Figure 4A). Our results showed that the BrdU^+^/DCX^+^ cells were markedly decreased in SAH + vehicle group, however, 17-AAG treatment notably increased the BrdU^+^ DCX^+^ cells 4 weeks after SAH (Figure 4B).

### Effects of 17-AAG on Pro-inflammatory, Neuroprotective and Pro-neurogenesis Protein Levels

**Figure 5:**
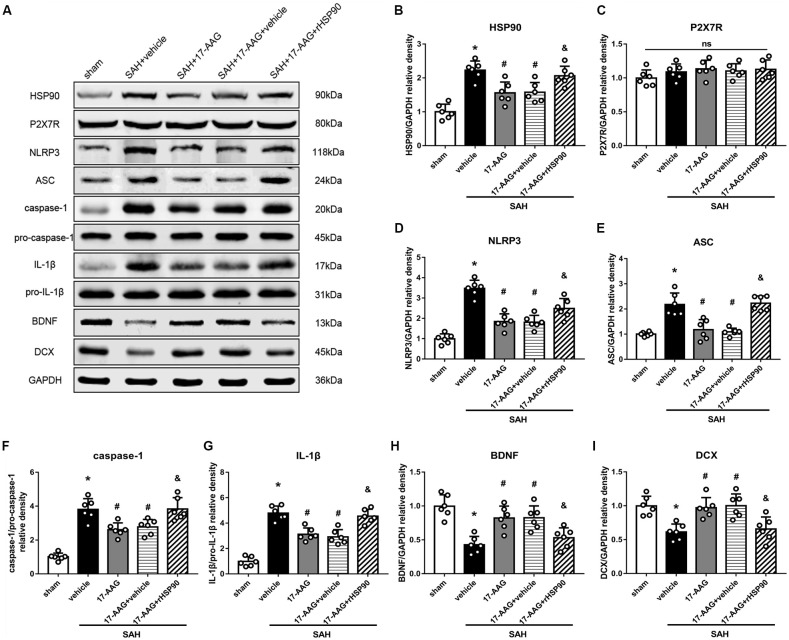
Effects of 17-AAG and recombinant HSP90 (rHSP90) on the expression of HSP90 and its downstream signaling proteins 48 h after SAH. **(A)** Representative western blots. **(B–I)** Quantitative analyses of HSP90, P2X7 receptor (P2X7R), NLRP3, ASC, caspase-1, interleukin-1β (IL-1β), brain-derived neurotropic factor (BDNF) and DCX in the left hemisphere; *n* = 6 per group. ns, no significant. *P < 0.05 vs. sham group; ^#^*P* < 0.05 vs. SAH + vehicle group; ^&^*P* < 0.05 vs. SAH + 17-AAG + vehicle. The error bars represent SD.

**Figure 6:**
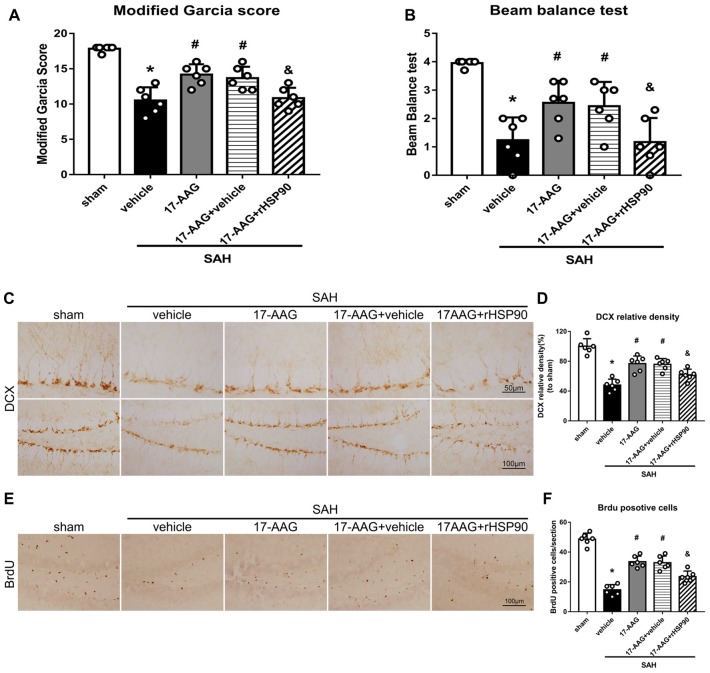
Effects of 17-AAG and rHSP90 on neurobehavior and neurogenesis 48 h after SAH. **(A)** Modified Garcia scores and **(B)** beam balance test scores; *n* = 10 per group. **(C)** Immunohistochemistry staining and **(D)** quantitative analyses of DCX in the left hemisphere. **(E)** Immunohistochemistry staining and **(F)** quantitative analyses of BrdU in the left hemisphere; *n* = 4 per group. *P < 0.05 vs. sham; ^#^*P* < 0.05 vs. SAH + vehicle; ^&^*P* < 0.05 vs. SAH + 17-AAG + vehicle. The error bars represent SD.

The NLRP3 inflammasome, assembled by NLRP3, ASC and caspase-1, play a vital role in the process of proinflammatory cytokine IL-1β maturation from pro- IL-1β. P2X7R has been shown to mediate NLRP3 inflammasome activations (Khalafalla et al., 2017). BDNF is an essential neurotropic factor for neurogenesis, and DCX employed as a marker of immature neurons, which was expressed exclusively in the early differentiation stage of neuronal stem cells. Forty-eight hours after SAH, the protein levels of HSP90, NLRP3, ASC, caspase-1 and IL-1β were dramatically increased compared with those in the sham group (Experiment 3). In contrast, the BDNF and DCX levels, as well as the number of DCX^+^ cells or BrdU^+^ progenitor cells, were significantly decreased in the SAH + vehicle group compared with those in the sham group (*P* < 0.05; Figures 5, 6). 17-AAG treatment (80 mg/kg) not only decreased the expression of HSP90, NLRP3, ASC, caspase-1 and IL-1β, but also increased the protein levels of BDNF and DCX, as well as the number of DCX^+^ cells (Supplementary Figure S4A) and BrdU^+^ progenitor cells in SAH mice compared to vehicle (*P* < 0.05; Figures 5, 6). Administration of rHSP90 reversed these 17-AGG-induced changes. The expression of P2X7R was not affected by 17-AAG treatment (*P* > 0.05, Figure 5C). In addition, the 17-AAG administration did not affect the proteins level of P2X7R, NLRP3, ASC, caspase-1, IL-1β, BDNF and DCX in sham condition (*P* > 0.05, Supplementary Figure S3).These results revealed that 17-AAG exerted neuroprotective effect by the effects of anti-inflammation of inflammasome and pro-neurogenesis.

### Intraventricular Injection of rHSP90 Enhances SAH-Induced Protein Changes and Neurological Deficits

**Figure 7:**
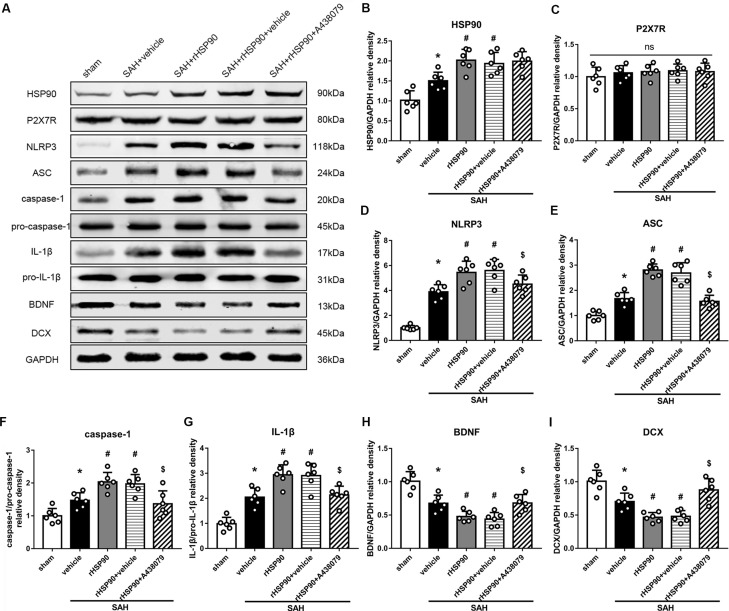
Effects of rHSP90 and P2X7R antagonist A438079 on the expression of HSP90 and its downstream signaling proteins 48 h after SAH. **(A)** Representative western blots. **(B–I)** Quantitative analyses of HSP90, P2X7R, NLRP3, ASC, caspase-1, IL-1β, BDNF and DCX in the left hemisphere; *n* = 6 per group. ns, no significant. *P < 0.05 vs. sham; ^#^*P* < 0.05 vs. SAH + vehicle; ^$^*P* < 0.05 vs. SAH + rHSP90 + vehicle. The error bars represent SD.

**Figure 8:**
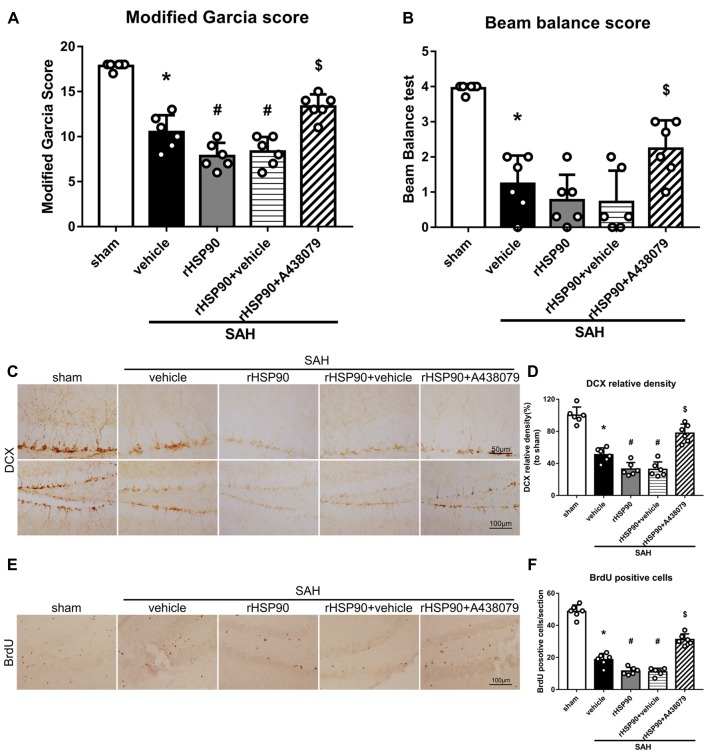
Effects of rHSP90 and A438079 on neurobehavior and neurogenesis 48 h after SAH. **(A)** Modified Garcia scores and **(B)** beam balance test scores; *n* = 10 per group. **(C)** Immunohistochemistry staining and **(D)** quantitative analyses of DCX in the left hemisphere. **(E)** Immunohistochemistry staining and **(F)** quantitative analyses of BrdU in the left hemisphere; *n* = 4 per group. *P < 0.05 vs. sham; ^#^*P* < 0.05 vs. SAH + vehicle; ^$^*P* < 0.05 vs. SAH + rHSP90 + vehicle. The error bars represent SD.

We used exogenous HSP90 to further confirm this pathway. The protein levels of HSP90, NLRP3, ASC, caspase-1 and IL-1β were dramatically increased, while those of BDNF and DCX, as well as the number of DCX^+^ cells (Supplementary Figure S4B) and the number of BrdU^+^ progenitor cells were significantly decreased, in the SAH + rHSP90 group compared with those in the SAH + vehicle group (*P* < 0.05; Figures 7, 8; Experiment 4). However, the specific P2X7R inhibitor A438079 reversed these effects of rHSP90. The expression of P2X7R was not changed (Figure 7C). The administration of rHSP90 markedly exacerbated the SAH-associated neurological deficits as assessed by the modified Garcia and beam balance tests 48 h after SAH (Figures 8A,B). Collectively, these results suggest exogenous HSP90 worsen neurobehavior, exacerbated the neurogenesis damage, as well as increased inflammation after SAH.

### P2X7R Inhibition Attenuates SAH-Induced Effects in the Hippocampus

**Figure 9:**
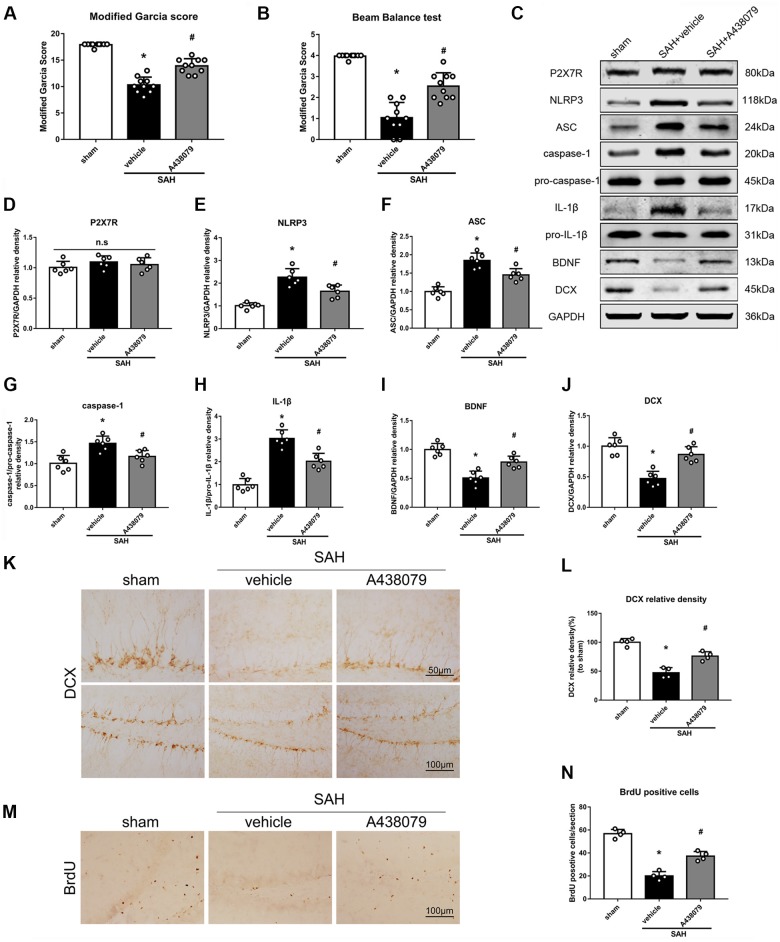
Effects of A438079 on the SAH-induced signaling pathway and neurogenesis 48 h after SAH. **(A)** Modified Garcia scores and **(B)** beam balance test scores; *n* = 10 per group. **(C)** Representative western blots. **(D–J)** Quantitative analyses of P2X7R, NLRP3, ASC, caspase-1, IL-1β, BDNF and DCX in the left hemisphere; *n* = 6 per group. **(K)** Immunohistochemistry staining and **(L)** quantitative analyses of DCX in the left hemisphere. **(M)** Immunohistochemistry staining and **(N)** quantitative analyses of *BrdU* in the left hemisphere; *n* = 4 per group. ns, no significant. *P < 0.05 vs. sham; ^#^*P* < 0.05 vs. SAH + vehicle. The error bars represent SD.

To further confirm that P2X7R is involved in the SAH-activated pathway, SAH mice were treated with A438079 (Experiment 6). As expected, A438079 ameliorated the SAH-induced neurological deficits (Figures 9A,B). The expression of NLRP3, ASC, caspase-1 and IL-1β was significantly decreased, while the BDNF and DCX protein levels and the number of BrdU^+^ progenitors were notably increased, in the SAH + A438079 group compared with those in the SAH + vehicle group (Figure 9).

### 17-AAG and A438079 Inhibit the NLRP3, ASC and Caspase-1 Interaction After SAH

**Figure 10:**
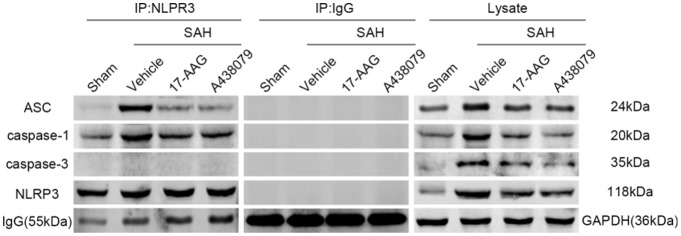
Effects of 17-AAG and A438079 on the SAH-induced NLRP3 inflammasome resemble. Immunoprecipitation (IP) assay of NLRP3: ASC, NLRP3: caspase-1 and NLRP3: caspase-3 in left hemisphere after SAH.

In the previous sections, we demonstrated that 17-AAG and A438079 can decreased the expression of NLRP3 inflammasome proteins. To further investigate the inhibition effect of 17-AAG or A438079 on NLRP3 inflammasome formation, the IP was performed to detect the interaction of NLRP3 with ASC and caspase-1. As a control, the IP between NLRP3 and caspase-3 also performed. The results (Figure 10) show that the interaction of NLRP3: ASC and NLRP3: caspase-1 was markedly increased in SAH+vehicle group compared to sham group, while they were reversed by 17-AAG or A438079 administration. These results suggested that the 17-AAG inhibited NLRP3 inflammasome formation by breaking the interaction of NLRP3: ASC and NLRP3: caspase-1.

## Discussion

**Figure 11:**
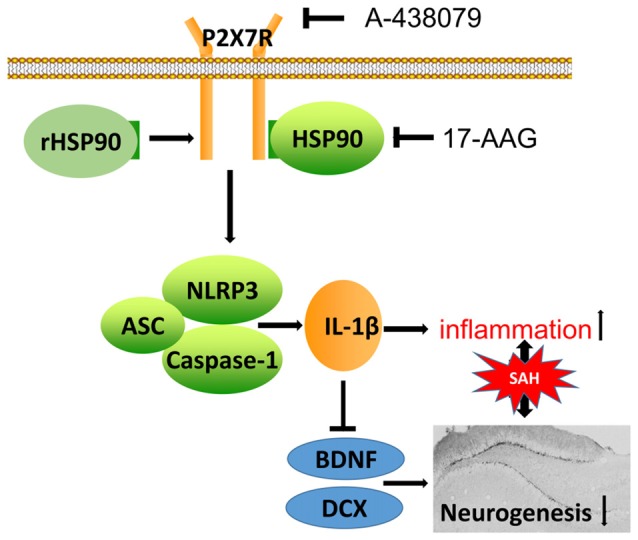
Potential mechanistic pathway by which 17-AAG regulates inflammation and neurogenesis after SAH. SAH damage causes HSP90 upregulation, which leads to P2X7R activation and stimulates NLRP3 inflammasome assembly. The resultant maturation of IL-1β is responsible for the inflammation and reparative neurogenesis. 17-AAG inhibits HSP90, resulting in attenuated inflammation and increased neurogenesis.

In the present study, we explored the effects of HSP90 modulation on inflammation and neurogenesis, as well as their potential mechanisms, after experimentally induced SAH in mice. Our results demonstrated that HSP90 inhibition with the specific inhibitor 17-AAG improved short- and long-term neurobehavioral outcomes, ameliorated brain edema, inhibited the NLRP3 inflammasome, and increased neurogenesis in the hippocampuses of SAH mice. Administration of 17-AAG was associated with downregulation of the P2X7R/NLRP3 inflammasome axis and IL-1β, and increased BDNF and DCX expression and the number of BrdU-positive cells after SAH. However, administration of rHSP90 reversed the beneficial effects of 17-AAG, worsened neurobehavioral functions, induced the NLRP3 inflammasome, and inhibited neurogenesis. A selective antagonist of P2X7R reversed the detrimental effects of rHSP90. Taken together, our results indicated that HSP90 inhibition with 17-AAG might ameliorate neuroinflammation via a mechanism at least in part mediated by the P2X7R/NLRP3 inflammasome pathway and increase neurogenesis after SAH (Figure 11).

HSPs, also known as chaperones, modulate protein folding and maturation, which are essential in the maintenance of cellular protein homeostasis. Previous studies have indicated that HSPs are involved in several neurological diseases, including Alzheimer’s disease and stroke (Bradley et al., 2014; Qi et al., 2015; Lackie et al., 2017). Additionally, it has been shown that HSPs are selectively overexpressed in atherosclerotic lesions and that the levels of anti-HSP antibodies are increased in patients with a history of stroke (Banecka-Majkutewicz et al., 2014). HSP90 is abundantly expressed in the central nervous system and plays an important role in the folding, maturation, stabilization and activation of its client proteins, contributing to inflammation and exerting a pronounced effect on inflammatory pathways (Tukaj and Wegrzyn, 2016; Khandia et al., 2017). A previous study reported that HSP90 was abundant in neurons as well as expressed by activated microglia in the brain (Jeon et al., 2004). Consistent with this study, our double immunofluorescence staining results showed that HSP90 was localized in neurons, microglia, and NPCs. Additionally, western blotting and immunohistochemistry demonstrated that the protein level of HSP90 was upregulated in the left hippocampus after SAH. Elevated HSP90 protein levels have been reported both in the sera of acute ischemic stroke patients and in the brain of an ischemic mouse model (Qi et al., 2015). In agreement with these reports, our results indicated that HSP90 protein expression was increased in the acute SAH phase. However, our IHC staining revealed a higher HSP90 expression in the GCL than that in the SGZ after SAH compared to shams. This may be explained as following: A previous *in vitro* study showed that the expression of HSP90 protein increased along neuronal differentiation induced by FK506 (Quintá et al., 2010). Therefore, we speculated that HSP90 genes may express in both GCL mature neurons and SGZ immature neurons at 24 h after SAH, but there were higher expressions of HSP90 in mature neurons (Figure 1D). The reduced numbers of DCX positive immature neurons after SAH also contributed the less cell numbers of HSP90 co-localization (Figure 2D). Future studies are needed to further elucidate the distribution patterns in details.

It has been suggested that inhibition of HSP90 exerts neuroprotective effects, including attenuation of the inflammatory response, protection of BBB integrity, prevention of neuronal cell death, and preservation of NPCs in various diseases, such as traumatic brain injury and ischemic stroke (Kim et al., 2012; Bradley et al., 2014; Qi et al., 2014; Tukaj and Wegrzyn, 2016; Wang et al., 2018). As a potent inhibitor of HSP90, 17-AAG binds HSP90 and inhibits its function, which eventually results in the degradation of the HSP90 client proteins. In addition, previous studies have reported that 17-AAG protects neural progenitors, inhibits neuronal cell death, and ameliorates the memory impairment caused by ischemic stroke (Bradley et al., 2014; Li et al., 2015). Therefore, we focused on the protective effects of 17-AAG in an experimental SAH model.

Brain edema, caused by inflammation and BBB disruption, is a main pathological cause of mortality and poor outcomes after SAH. In the present study, 17-AAG treatment reduced SAH-induced neurological deficits and brain edema 48 h after SAH surgery. Moreover, the functions of sensorimotor coordination and balance, as well as cognitive and memory abilities, as assessed by the Rotarod and Morris water maze tests, respectively, were improved by 17-AAG treatment in our study of long-term outcomes. Taken together, our results indicate that the inhibition of HSP90 can potentially improve short- and long-term outcomes of SAH.

Neurogenesis plays an essential role in recovery after SAH (Tian et al., 2017), and has been shown to continuously occur in the SGZ of the hippocampal DG. BDNF plays an essential role in neurogenesis, including cell survival, synaptic plasticity, and progenitor cell differentiation (Chao et al., 2006). DCX, which is only expressed in the early stage of differentiation, serves as a marker of newly generated neurons in the adult DG (Spampanato et al., 2012). In addition, DCX is expressed by neuronal progenitor cells and is important for neuronal migration, a process critical for brain development and recovery from injury (Kawauchi, 2015). Importantly, neurogenesis is closely related to BDNF and DCX, which are associated with neurological function, including memory (Moon et al., 2016).

Recently, inflammatory cytokines, particularly IL-1β, have been shown to be key modulators of neurogenesis (Ryan et al., 2013; Borsini et al., 2015). Therefore, inflammation and neurogenesis may be involved in the mechanism of the neuroprotective effects of HSP90 inhibition. One of the main inflammatory responses induced by SAH is the assembly of the NLRP3 inflammasome, which has been demonstrated to play a role in the pathophysiology of EBI (Zhou et al., 2017, 2018). The NLRP3 inflammasome is activated by three key proximal upstream modulators: reactive oxygen species, potassium and chloride (Tang et al., 2017). Once activated, the NLRP3 inflammasome mediates pro-caspase-1 cleavage to yield active caspase-1 and promote IL-1β maturation, subsequently contributing to inflammation. P2X7R activation has been demonstrated to induce NLRP3 inflammasome assembly and the maturation and release of IL-1β (Lister et al., 2007; Chen et al., 2013; Khalafalla et al., 2017). Moreover, a previous study reported that HSP90 was essential for the stabilization and function of P2X7R (Migita et al., 2016). In addition, HSP90 was recently shown to be one of the 11 molecules integrated in the P2X7R protein complex responsible for the activation of pain signaling cascades (Adinolfi et al., 2003; Nascimento et al., 2018). However, neither the mRNA nor protein level of P2X7R changes after HSP90 inhibitor administration (Migita et al., 2016; Nascimento et al., 2018). Consistent with these reports, our western blotting results demonstrated that 17-AAG treatment had no influence on the protein level of P2X7R, while it did downregulate the SAH-induced expression of NLRP3, caspase-1 and IL-1β, upregulate that of BDNF and DCX, and increase the number of BrdU-positive cells. To further validate this pathway, rHSP90 and A438079 were applied to investigate the effects of SAH on neurological functions and the expression of downstream proteins. Our results showed that rHSP90 worsened the SAH-induced neurobehavioral deficits, increased the expression of NLRP3, caspase-1 and IL-1β, decreased the expression of BDNF and DCX, and reduced the number of BrdU-positive cells. Pretreatment with A438079, a potent antagonist of P2X7R, reversed these changes. Interestingly, the expression of P2X7R, as evaluated by western blotting, showed no significant change after rHSP90 administration, which indicated that HSP90 regulates P2X7R activity rather than the protein level. Furthermore, the interaction between NLRP3: ASC and NLRP3: caspase-1 was inhibited by the 17-AAG or A438079 administration. Together, our findings suggest that HSP90/P2X7R/NLRP3 inflammasome pathway involved in the inflammatory response and such proinflammatory microenvironment contributed to the impaired neurogenesis after SAH. The inhibition of HSP90 with 17-AAG alleviated inflammation through the P2X7R/NLRP3 inflammasome pathway and increased neurogenesis. Therefore, inhibition of HSP90 with 17-AAG may be a promising therapeutic strategy for the management of SAH.

Our study has some limitations. First, since we focused on the anti-inflammatory and neurogenesis-promoting effects of 17-AAG after SAH, we cannot exclude the possibility that HSP90 inhibition may also exert other protective effects, such as the amelioration of autophagic death and preservation of BBB integrity (Li et al., 2015; Qi et al., 2015). Second, it is well known that neural stem/progenitor cells, mostly located in the SVZ and SGZ, proliferate and migrate during recovery from brain injuries in some experimental stroke models (Nakayama et al., 2010). However, we only focused on neurogenesis in the hippocampus. Third, a previous study reported that inhibition of HSP90 alleviated IκB/NF-κB-mediated inflammation (Qi et al., 2014). Therefore, it is possible that other pathways may also contribute to the reduction of inflammation and enhancement of neurogenesis induced by the inhibition of HSP90. Further studies are required to fully elucidate the mechanism of the neuroprotective effects of 17-AAG involving HSP90. In addition, the HSP70 was also increased by 17-AAG administration in our results consistent to the previous findings in neurodegenerative mice model (Waza et al., 2005). The neuroprotective mechanism of 17-AAG through HSP70 need to be explored in future.

## Conclusion

Our study demonstrated that inhibition of HSP90 with 17-AAG improves neurological functions, ameliorates brain edema, as well as attenuates neuroinflammation through the P2X7R/NLRP3 inflammasome signaling pathway and induce neurogenesis-based recovery in a mouse model of experimental SAH. Therefore, inhibition of HSP90 with 17-AAG may be a promising therapeutic strategy in SAH management.

## Author Contributions

YZ and FeL conceived and designed the experiments. YZ, JW, and FaL performed the experiments. YZ and JL collected and analyzed the data. YZ and XY drafted the manuscript. YZ and LH revised the manuscript.

## Conflict of Interest Statement

The authors declare that the research was conducted in the absence of any commercial or financial relationships that could be construed as a potential conflict of interest.

## Supplementary Material

FIGURE S1 Experimental design and the number of mice used in each group. **(A)** Designs of Experiments 1–6. **(B)** Numbers of mice used and mortality in each group. *Animals shared with the sham group in experiment 1; ^#^animals shared with the SAH + vehicle group in Experiment 3.

FIGURE S2 Expression of HSP70 and HSP40 after subarachnoid hemorrhage (SAH) and affected by 17-AAG treatment. **(A)** Representative western blot images and quantitative analyses of the **(B)** HSP70 and **(C)** HSP40 time course in the left hemisphere after SAH. **(D)** Representative western blot images and quantitative analyses of the **(E)** HSP70 and **(F)** HSP40 after 17-AAG treatment in the left hemisphere after SAH. The expression of HSP70 increased after SAH, and administration of 17-AAG can further increased the HSP70 protein level. However, the HSP40 protein level showed no significant difference after SAH and not affected by 17-AAG treatment. *n* = 6 per group. ns, no significant. **P* < 0.05 vs. sham; ^#^*P* < 0.05 vs. SAH + vehicle. The error bars represent SD.

FIGURE S3 Effects of 17-AAG in the pathway in sham condition. **(A)** Representative western blots. **(B–H)** Quantitative analyses of P2X7R, NLRP3, ASC, caspase-1, IL-1β, BDNF and DCX in the left hemisphere in the sham condition; the expression of P2X7R, NLRP3, ASC, caspase-1, IL-1β, BDNF and DCX show no significant difference among sham, sham + vehicle and sham + 17-AAG group. *n* = 6 per group; n.s, no significant.

FIGURE S4 DCX positive cells count in the left hemisphere in the Experiments 3 and 4. **(A)** DCX positive cells count in the Experiment 3; *n* = 4 per group. **P* < 0.05 vs. sham; ^#^*P* < 0.05 vs. SAH + vehicle; ^$^*P* < 0.05 vs. SAH + 17-AAG + vehicle. The error bars represent SD. **(B)** DCX positive cells count in the Experiment 4; *n* = 4 per group. **P* < 0.05 vs. sham; ^#^*P* < 0.05 vs. SAH + vehicle; ^&^*P* < 0.05 vs. SAH + 17-AAG + vehicle; ^$^*P* < 0.05 vs. SAH + rHSP90 + vehicle.
