# Supplementary figures and images for: Inhibition of Heat Shock Protein 90 by 17-AAG Reduces Inflammation via P2X7 Receptor/NLRP3 Inflammasome Pathway and Increases Neurogenesis After Subarachnoid Hemorrhage in Mice
Source: Front Mol Neurosci. 2018 Nov 6;11:401. doi: 10.3389/fnmol.2018.00401 (PMC6232389; doi:10.3389/fnmol.2018.00401)

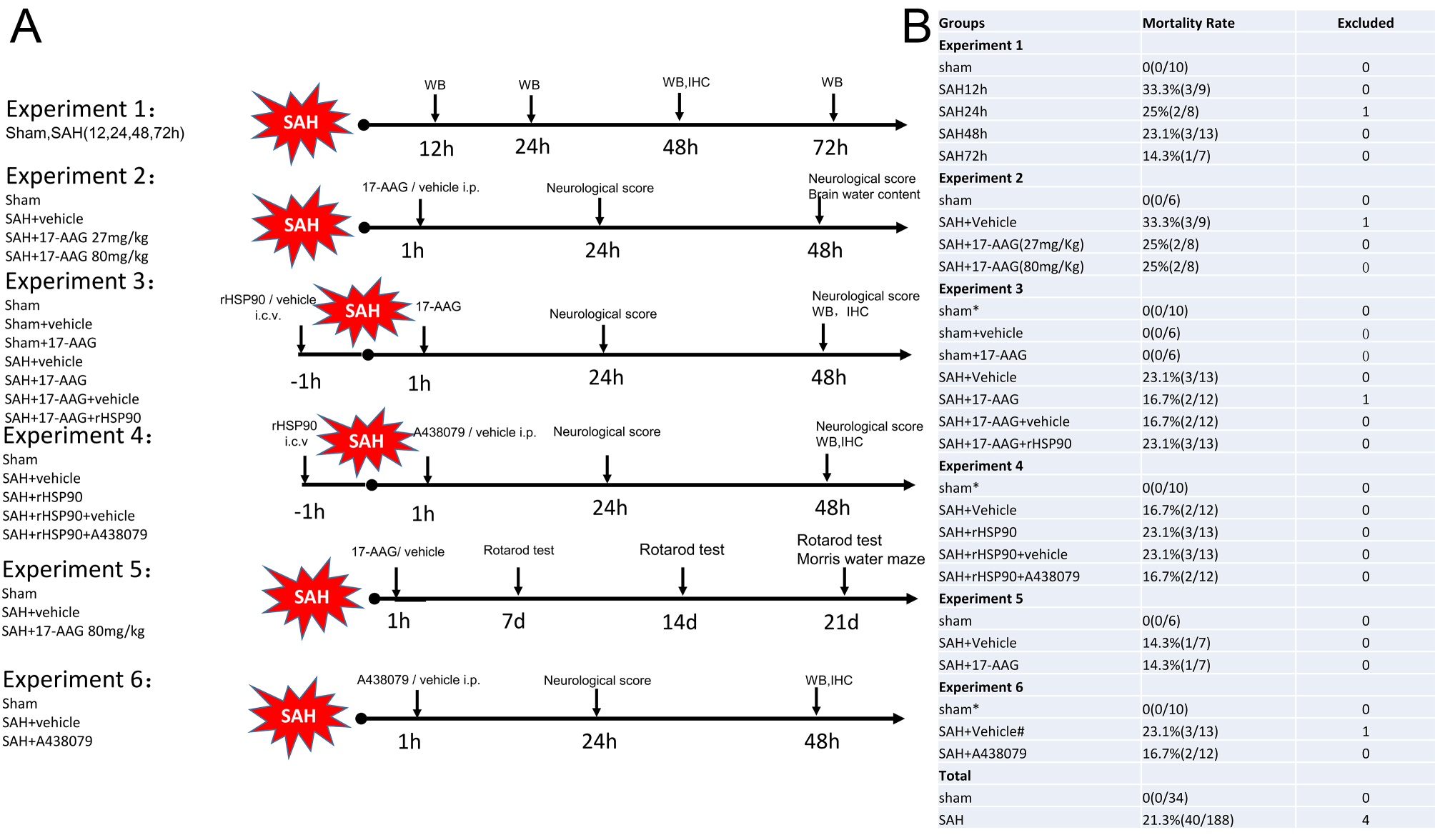

Supplement: FIGURE S1 — Experimental design and the number of mice used in each group. (A) Designs of Experiments 1–6. (B) Numbers of mice used and mortality in each group. *Animals shared with the sham group in experiment 1; #animals shared with the SAH + vehicle group in Experiment 3. [file Image_1.TIF]

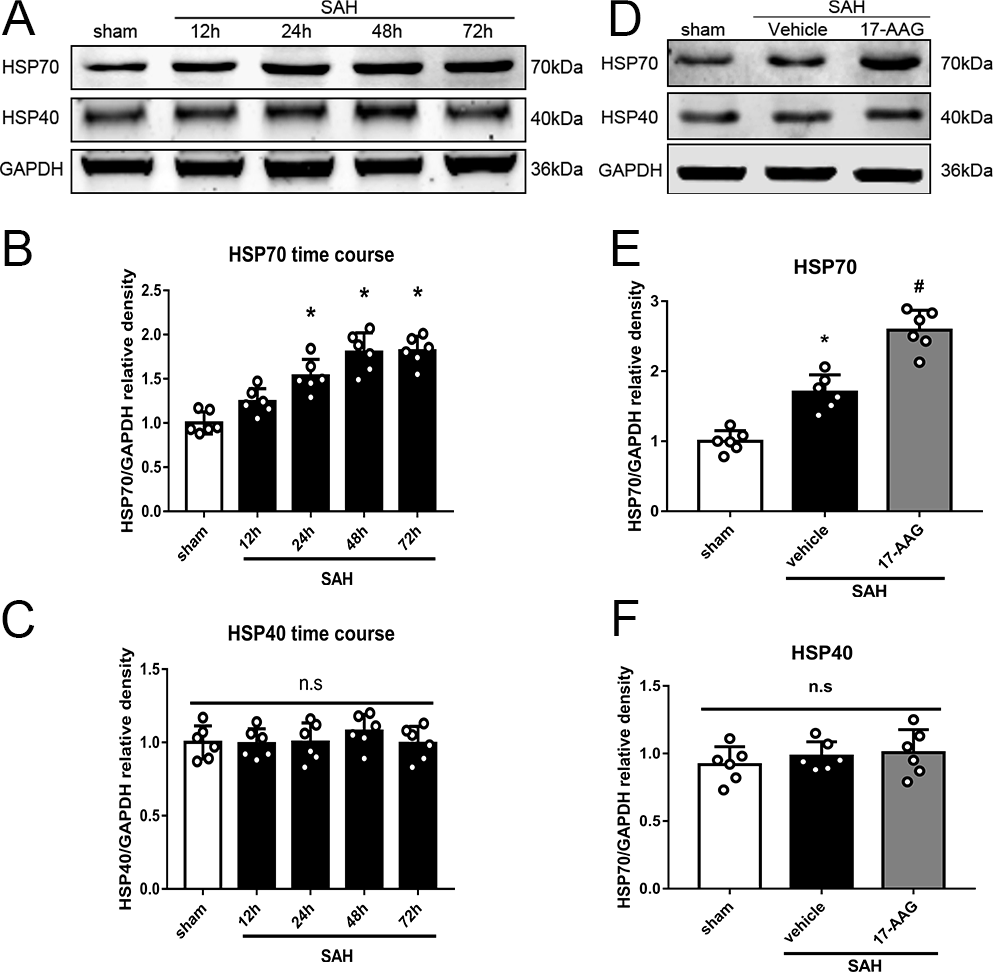

Supplement: FIGURE S2 — Expression of HSP70 and HSP40 after subarachnoid hemorrhage (SAH) and affected by 17-AAG treatment. (A) Representative western blot images and quantitative analyses of the (B) HSP70 and (C) HSP40 time course in the left hemisphere after SAH. (D) Representative western blot images and quantitative analyses of the (E) HSP70 and (F) HSP40 after 17-AAG treatment in the left hemisphere after SAH. The expression of HSP70 increased after SAH, and administration of 17-AAG can further increased the HSP70 protein level. However, the HSP40 protein level showed no significant difference after SAH and not affected by 17-AAG treatment. n = 6 per group. ns, no significant. *P < 0.05 vs. sham; #P < 0.05 vs. SAH + vehicle. The error bars represent SD. [file Image_2.TIF]

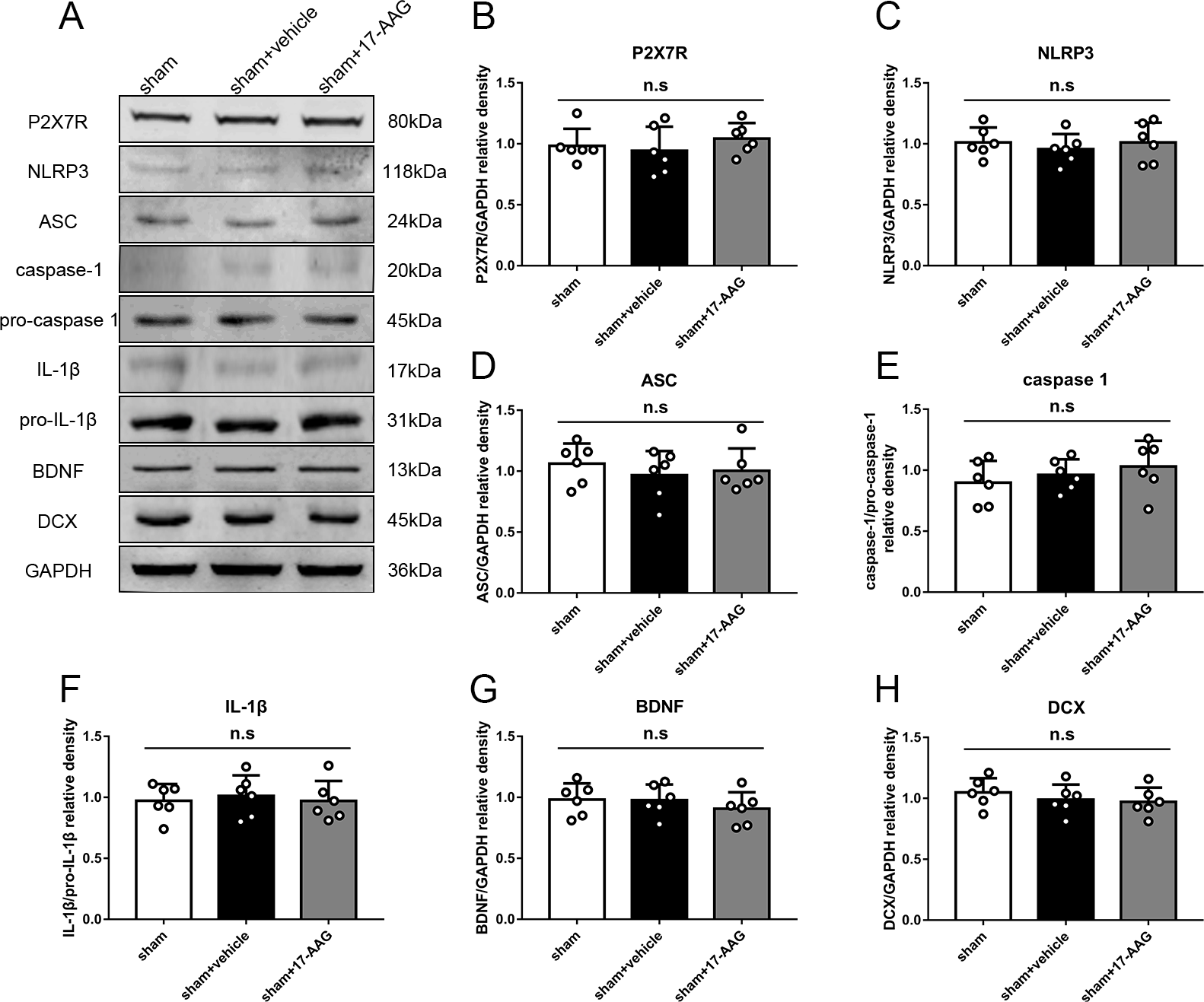

Supplement: FIGURE S3 — Effects of 17-AAG in the pathway in sham condition. (A) Representative western blots. (B–H) Quantitative analyses of P2X7R, NLRP3, ASC, caspase-1, IL-1β, BDNF and DCX in the left hemisphere in the sham condition; the expression of P2X7R, NLRP3, ASC, caspase-1, IL-1β, BDNF and DCX show no significant difference among sham, sham + vehicle and sham + 17-AAG group. n = 6 per group; n.s, no significant. [file Image_3.TIF]

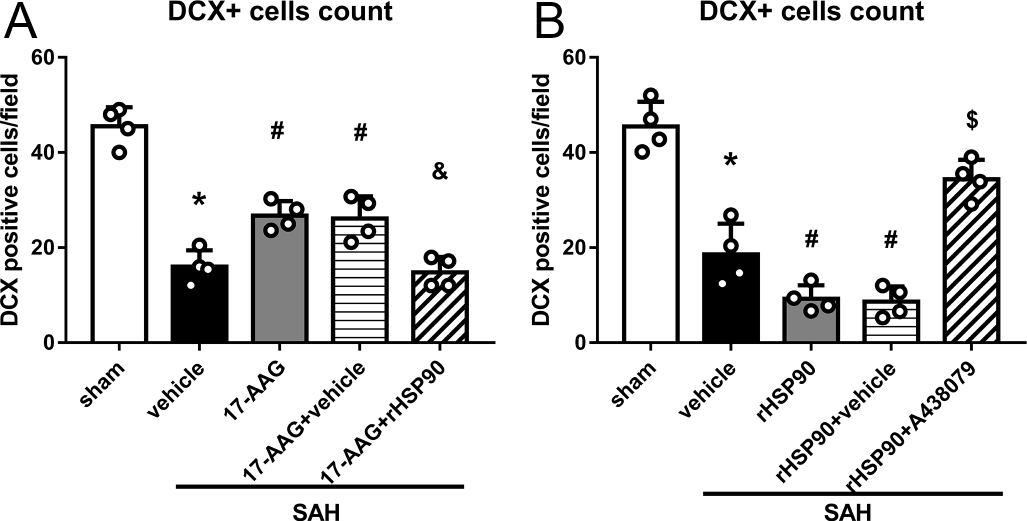

Supplement: FIGURE S4 — DCX positive cells count in the left hemisphere in the Experiments 3 and 4. (A) DCX positive cells count in the Experiment 3; n = 4 per group. *P < 0.05 vs. sham; #P < 0.05 vs. SAH + vehicle; $P < 0.05 vs. SAH + 17-AAG + vehicle. The error bars represent SD. (B) DCX positive cells count in the Experiment 4; n = 4 per group. *P < 0.05 vs. sham; #P < 0.05 vs. SAH + vehicle; &P < 0.05 vs. SAH + 17-AAG + vehicle; $P < 0.05 vs. SAH + rHSP90 + vehicle. [file Image_4.TIF]
